# Getah Virus in Review: An Emerging Zoonotic Arbovirus

**DOI:** 10.1155/tbed/9245726

**Published:** 2026-04-07

**Authors:** Zhengyi Qiu, Duo Zhang, Qingmiao Fan, Zhihao Hu, Haobin Xie, Nan Li, Pengpeng Xiao

**Affiliations:** ^1^ Wenzhou Key Laboratory for Virology and Immunology, Institute of Virology, Wenzhou University, Wenzhou, 325035, China, wzu.edu.cn; ^2^ College of Veterinary Medicine, Jilin University, Changchun, 130062, China, jlu.edu.cn

## Abstract

Getah virus (GETV), a zoonotic virus, belongs to *Togaviridae*, *Alphavirus*. GETV leads to diarrhea in piglets and dysfunction of the reproductive system in sows, with pregnant sows potentially experiencing abortion, fetal death, or weak offspring, causing a severe impact on the livestock industry and resulting in economic losses. Currently, multiple instances of cross‐host transmission of GETV are occurring. There has been no systematic, comprehensive summary of the cross‐species transmission characteristics of GETV. Research on the infection mechanism may be insufficient to elucidate it. Therefore, further researches are needed. And the potential vaccine for GETV treatment is still under development, even though there were some therapies to alleviate the harms that GETV brings. In this paper, the temporal and spatial distribution, disease outbreak, transmission route, cross‐species transmission, and interventions of GETV are reviewed, aiming at providing a reference guide to aid public health personnel involved in GETV research and the prevention of GETV.

## 1. Introduction

**Figure 1:**
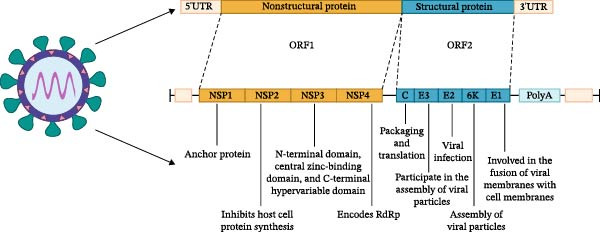
The genetic structure of the GETV. The GETV genome contains two open reading frames, ORF1 and ORF2, which encode nonstructural proteins and structural proteins, respectively. The four nonstructural proteins are mainly responsible for viral transcription, replication, and regulation of the host cell response. The five structural proteins are mainly responsible for the composition of the viral particle and participate in assembly, protection, and invasion of the host cell.

Getah virus (GETV), a single‐stranded, positive‐sense RNA virus, belongs to the *Alphavirus* within the *Togaviridae* [1]. GETV is a constituent of the Semliki Forest virus (SFV) antigenic complex, and several human pathogens, such as O’nyong‐nyong virus (ONNV), Chikungunya virus (CHIKV), and Ross River virus (RRV), are also included [2]. The full‐length viral genome is between 11,000 and 12,000 nucleotides [3]. The 5′ end possesses a methylated (7‐methylguanosine) cap structure, while there is a variable number of poly (A) tails at the 3′ end [4]. For GETV, it contains two open reading frames (ORFs) encoding structural and nonstructural proteins, respectively. These proteins have been shown to play distinct roles in the process of viral replication. And the first ORF encoding nonstructural proteins Nsp1, Nsp2, Nsp3, and Nsp4 is located at the 5′ end [5]. For the second ORF encoding the structural proteins capsid (C), E3, E2, 6K, and E1 is found at the 3′ end (Figure 1) [6]. According to the E2 gene sequence, GETV is divided into four different genotypes, namely, GI, GII, GIII, and GIV [7]. The initial isolate MM20021 from mosquitoes in Malaysia is the only strain in Group I. Both the Sagiyama virus strain and the M6‐Mag132 virus strain from Japan in 1956 are in Group II. The virus strains LEIV16275Mag, GETV/SW/Thailand/2017, YN12031, and the new virus strain Rbsq202206 are in Group IV. Group IV represents a novel evolutionary group that emerged three decades ago, and YN12031 is the representative strain. Nowadays, the major strains of GETV isolated from pigs belong to Group III. Among these genotypes, the GIII group is the most prevalent at present, which poses a grave threat to animal health and food safety and causes economic losses.

After MM2021 was first isolated in 1955 [8], the detection of GETV in numerous countries such as China, Japan, Thailand, Mongolia, Philippines, Korea, India, Russia, and Australia has been reported. As a zoonotic virus, mosquitoes such as *Culex* and *Aedes* are the primary vectors, and an increasing number of host reservoir species like horses, pigs, monkeys, and even humans are found to carry GETV. Within the species mentioned before, the main susceptible hosts are horses and pigs [9]. The horses infected with GETV will exhibit skin eruptions, fever, and limb edema. GETV leads to anorexia, fever, depression, diarrhea, and reproductive disorders in infected pigs [10]. GETV remains infectious under various environmental conditions, making it easy to spread to susceptible hosts. In recent years, with the rapid spread of *Aedes*, the circulating territories of GETV have expanded. Moreover, GETV‐neutralizing antibodies were present in humans and cattle [11], indicating that there are potential public health risks and pose a growing threat to the animal industry. Although GETV is closely related to RRV, which can result in human diseases, the consequences of its infection are still unclear. Besides the rising number of novel GETV strains from insects and mammal animals, the seroepidemiological results of multiple studies showed that humans may have been exposed to the virus, highlighting the potential risks to public health, particularly the emerging strains that may be pathogenic. Other alphaviruses and GETV may possess shared vectors, leading to infection with different viruses. The situation may cause complicated clinical symptoms and increase public health risks. With the distribution of GETV, the risks in public health and husbandry are increasing. It is urgent and necessary to pay attention to block its transmission and develop novel interventions.

## 2. Pathogenesis and Replication Mechanisms

The virions of GETV are spherical particles with an approximate diameter of 70 nm and consist of three parts: the capsid, inner viral core, and the outer envelope adorned with glycoproteins [12]. A linear genomic RNA molecule and 240 copies of the CPs make up the viral core [13]. Each trimeric spike has three copies of the heterodimers E1/E2 or heterotrimers E1/E2/E3, and 80 trimeric spikes make up the outer envelope. And the heterodimers are immobilized in the membrane. Three heterodimers form a virus spike that is crucial for GETV to attach to the cell surface and enter the cell [14].

There are four distinct categories of nonstructural proteins in GETV. Nsp1, a methyltransferase, binds to the cell membrane. Protease, viral helicase, and a hypothetical C‐terminal methyltransferase domain are all present in Nsp2, which has the capacity to bind to a variety of host proteins, thus effectively inhibiting protein synthesis in host cells. Nsp3, a kind of phosphoprotein, recruits host factor G3BP, subsequently hindering the development of cellular stress granules. Nsp4 functions as the viral RNA‐dependent RNA polymerase. Among the nonstructural proteins, Nsp3 is related to the viral pathogenicity, and the absence of Nsp3 reduces the virulence [15]. The nonstructural proteins are crucial for replicating, translating, and evading the host immune response.

E1, the type I transmembrane protein, exerts an effect on immunogenicity and host range [2]. Besides, three different structural domains: a cytoplasmic tail, a transmembrane helix (TM), and an ectodomain are included in E1 [16]. Apart from being a structural component, the primary function during viral entry is to facilitate the fusion of the viral membrane with the cell membrane [17].

E2 is also a type I transmembrane protein and functions to bind to the receptor. E2, a principal antigen of *alphaviruses*, can connect to host cell receptors. The capsid offers a physical protective encasement for viral genomic RNA and is also involved in the replication [18]. In addition, the connection between E1 and E2, capsid and E2 is crucial for viral germination, highlighting the significance of E2 in the replication of GETV [19]. E2 and E1 together form the virus vesicle membrane, entering into the host cell by endocytosis, subsequently fusing through adhesion factors present on the membrane. There are two domains (C‐terminal and N‐terminal) in the capsid protein. The C‐terminal one is a conserved protease, which is responsible for cracking capsids in polyproteins. The N‐terminal one exhibits an obvious positive charge that binds to the viral RNA packaging signal (PS) to ensure the smooth binding of viral RNA [20].

E3 is a protein consisting of 64 amino acids that plays a role in regulating the proper folding with PE2 and controlling the function of spikes together with the E1 protein [21]. Following the partial autolysation of early capsid proteins from polymeric structural proteins during translation, the E3 acts as a signal sequence to translocate the E3‐E2‐6K‐E1 complex into the endoplasmic reticulum (ER). The process facilitates the formation of the heterodimer with E2 and E1, playing a crucial role in the maturation of subsequent structural proteins [22].

6K protein is a small molecule polypeptide, which consists of 61 amino acids. Its synthesis follows the combination of E2/E1 dimer, and it is transported to the vicinity of the cell membrane for virus assembly [23]. 6K protein is associated with the replication, assembly, and virus budding and is considered the virus pore protein of GETV, which can mediate some ion transport and promote virus budding [24]. Some studies have shown that 6K protein is indispensable for GETV, and the mutation or deletion of the 6K gene will result in a substantial reduction in viral particle output and may even make GETV lose its ability to infect [25, 26].

An evolutionarily conserved low‐density lipoprotein receptor (LDLR) serves as a novel cellular entry factor [27]. The ectopic expression of LDLR contributes to the cellular binding and internalization, which is mediated by the connection between the E2‐E1 spike protein and the ligand‐binding domain (LBD) of LDLR. In addition, some essential amino acids were found to play an important part in viral entry. After the specific mutation of CR4 and CR5 domains, the virus’s ability to enter cells decreased by more than 20‐fold, suggesting targeting LDLR‐LBD may be a viable technique to develop antiviral drugs [28].

Each structural and nonstructural protein possesses different functions. Among them, the E2 exhibits high structural stability and has the GETV pathogenicity gene marker, making it suitable for clinical detection technologies [29]. The E2 surface contains antigenic determinants that can stimulate the body to produce antibodies, thus forming an antigen‐antibody relationship between the GETV and the host [30]. Besides, the E2 plays a pivotal role in the adsorption, infection, and the induction of host immune responses [31]. However, more research on GETV is needed to uncover the virus’s pathogenicity and mechanisms of cross‐species transmission.

**Figure 2:**
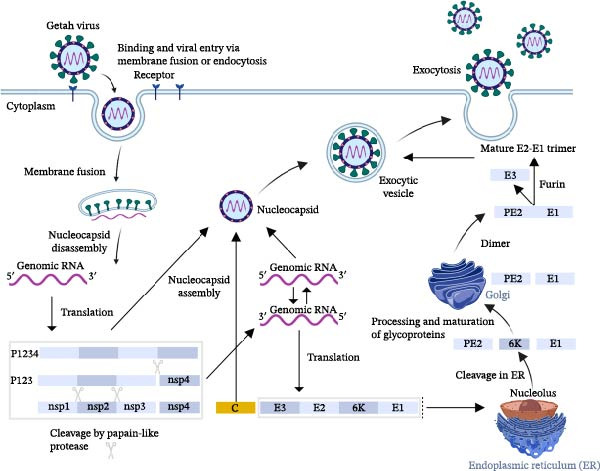
Replication cycle of GETV. The replication cycle begins with the binding of the virus particle to the receptor. Subsequently, the cell receptor mediates endocytosis, the fusion of the viral envelope, and the release of the genomic RNA. Then, the proteins are translated and processed. The glycoprotein is transported through the endoplasmic reticulum, undergoes processing in the Golgi apparatus, and is transported to the plasma membrane, where it finally binds to the nucleocapsid protein and is released through exocytosis.

When GETV invades cells, E2 first connects to cellular receptors and introduces virus particles into cells by endocytosis to form endosomes containing the virus [32]. In the endosome, as the pH value in the cavity gradually decreases to a certain threshold, the virus fuses with the cell membrane and releases the nucleocapsid core into the cytoplasm [33]. In the whole process, E1‐E2 dimer dissociates, E1 is inserted into the target membrane, and then E1 homotrimer is formed. The Cap protein disassembles the release virus genome, which is then translated utilizing P123 and P1234 as precursors. The polyprotein P1234 was broken into NS123 and NS4. And the nonstructural protein polypeptide is translated with genomic RNA as a template [34]. The function of nonstructural proteins is genome replication. An initial replication complex, built by NS123 and NS4 polypeptides, first forms a complete polypeptide chain that is used to synthesize the negative‐strand RNA [35]. It has been established that positive genomic 49S RNA and subgenomic 26S RNA are synthesized with the template negative‐strand RNA. The 26S RNA is then converted to the Cap‐pE2‐6K‐E1 polypeptide. Subsequently, serine proteases cleave the Cap protein from the polypeptide, while the process of cleavage of PE2‐6K‐E1 occurs within the ER to form pE2, 6K, and E1. Serine proteases cleave the Cap protein from the peptide, whereas PE2‐6K‐E1 is cleaved in the ER into pE2, 6K, and E1. Under the action of the GETV replication complex, subgenomic RNA was synthesized in large quantities, and the host cells were used as templates to translate structural proteins. These glycoproteins were transferred to the Golgi apparatus through vesicles. During this period, pE2 was cleaved into glycoproteins E3 and E2 by furan protease, then formed heterodimers with E1, and finally transferred to the plasma membrane [36]. The capsid recognizes the newly transcribed viral RNA, interacts with E1‐E2 heterodimers on the plasma membrane, assembles into a complete nucleocapsid, and forms mature progeny virus particles, which are released by budding (Figure 2) [37].

## 3. Vector and Susceptible Animals

GETV has been transmitted in various mosquitoes, such as *Culex vishnui*, *Culex tritaeniorhynchus*, *Culex fuscocephala*, *Anopheles vagus*, *Armigeres subalbatus*, *Anopheles sinensis*, *Aedes aegypti*, *Aedes albopictus*, *Aedes vexans nipponii*, and *Mansonia annulifera*, as well as in some unclassified mosquito species [38]. As to *Cx. tritaeniorhynchus* and *Ar. subalbatus*, both of them are widespread across Southeast Asia, and massive GETV isolation has been found in both species. It is noted that *Cx. tritaeniorhynchus* is considered the main carrier of GETV among livestock in Japan [39]. For *Ae. albopictus* brings about global attention because of its high invasion and vectorial ability in spreading lots of *arboviruses*. Therefore, in prevalent areas, the number, the distribution range, their susceptibility, and their role in the spread of *arboviruses* need more investigations. Three GETV strains have been isolated from *Culicoides* in Yunnan and Xinjiang in China, suggesting that *Culicoides* may also be a potential vector of GETV. Compared with mosquitoes, *Culicoides* is more abundant in nature, more widely distributed, and smaller in size, which makes it more difficult to detect. Therefore, its potential harm as a vector of GETV should be paid more attention to.

The transmission routes of GETV are diverse and complex, among which mosquito‐borne transmission is dominant [38]. In addition to the mosquito‐borne route, there are other transmission mechanisms of GETV. The virus was detected in the cerebral cortex of newborn piglets and aborted fetuses, indicating that GETV has the ability to vertically transmit across the placenta [40, 41]. At the same time, aerosol transmission in a high viral load environment poses an important risk, and inhalation of virus‐containing particles can lead to infection [42]. GETV may be transmitted by direct contact with nasal secretions in infected horses. Under the experimental conditions, a large amount of virus was detected in nasal secretions of horses with GETV (http://www.cfsph.iastate.edu/pdf/shic-factsheet-getah-virus).

GETV can infect many species, such as birds, pigs, foxes, rats, cattle, horses, kangaroos, and reptiles. Infected horses are mainly characterized by lymphadenopathy, rash, edema, and fever. For newborn piglets, GETV infection results in diarrhea, hind limb paralysis, and depression and, in some cases, often leads to death [43]. In pregnant sows, GETV leads to abortion and vertical transmission to the offspring. Occasionally, the infected piglets will first develop fever and anorexia and then present ataxia and tremor [44]. In nature, because pigs can produce high viremia titers and maintain the transmission cycle of the virus, they are the main amplification hosts of GETV. Additionally, GETV infects wild animals, causing fever, anorexia, neurological symptoms, and even death [45]. In 2017, 25 blue foxes in a farm in Shandong Province presented symptoms of fever, anorexia, and depression, and some suffered from neurological symptoms and died [46, 47]. Besides, the research report found that there are neutralizing antibodies in the serum, indicating that GETV has the potential to induce specific human diseases. It has been proven that GETV affects the reproductive system of male animals, causing the reduction of offspring, testicular damage, and the decrease of sperm quality and quantity [48]. It is worth noting that horses and pigs are the main storage hosts of GETV, while rabbits and rodents show higher susceptibility. Under experimental conditions, GETV can infect rabbits, mice, and so on (Figure 3B), which causes muscle inflammation, growth retardation, and even fatal encephalitis [49]. GETV proliferates in mosquitoes and spreads GETV by bites, while infected animals become virus storage hosts, and uninfected mosquitoes can obtain GETV. Therefore, there is a mosquito‐vertebrate host‐mosquito transmission cycle of GETV in nature (Figure 3A).

Figure 3Transmission modes of GETV and newly discovered hosts. (A) Transmission modes of GETV. In nature, the transmission of GETV has formed a mosquito‐vertebrate host‐mosquito transmission cycle. The transmission modes include mosquito‐borne transmission, vertical transmission, aerosol transmission, and direct contact (nasal secretions) transmission. The arrows between mosquitoes indicate that mosquitoes transmit GETV among themselves by biting hosts. (B) Newly discovered hosts of GETV. GETV has been identified as a zoonotic virus. Pigs and horses are the earliest hosts to show clinical symptoms after being infected with GETV. Pigs, horses, and cattle are the natural hosts of GETV. Subsequently, new hosts such as blue foxes, kangaroos, dogs, red pandas, pangolins, sheep, chickens, and birds have been discovered. In the laboratory, it has been found that mice and rabbits can be infected with GETV.

**Figure:**
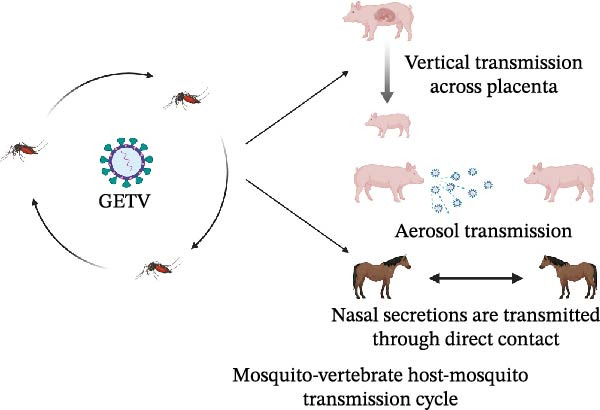
(A)

**Figure:**
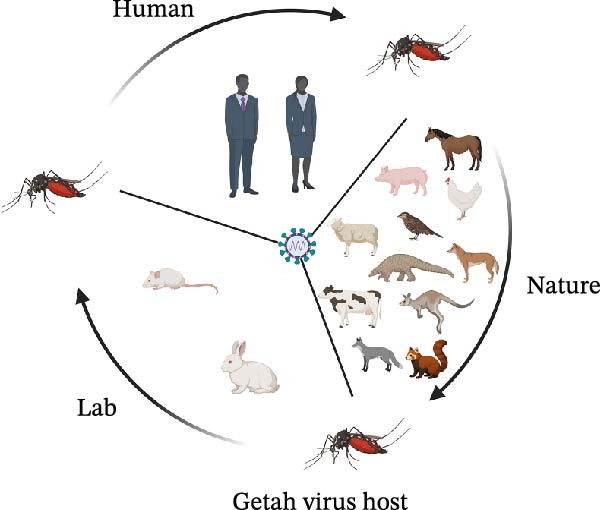
(B)

## 4. Geographical Spread

Nowadays, there are a large number of countries reporting the identification and the outbreak of GETV. Since 1955, the geographical distribution coverage has extended from 1° North to 60° North and from 38° East to 140° East [50]. More than 20 years after the isolation of GETV in Malaysia, Japan reported the virus found in horses and pigs in the 1970s and 1980s, respectively [51, 52]. With the serological finding of infected wild boars, the original outbreak of GETV might have taken place in 2012 and spread across Japan. From 2014 to 2016, some instances of GETV infection in racehorses were spotted in Japan. And in China, the first GETV strain was isolated in Hainan Province in 1964 [53, 54]. Then, the first infection in horses was detected in Guangdong Province in 2018 [55, 56]. And in Taiwan, the first outbreak of infected pigs happened in 2002. Subsequently, it was found in Henan, Anhui, Guangdong, Hubei, Sichuan, Fujian, Hunan, Shandong, Jiangsu, and Shanxi provinces (Figure 4B). Recently, lots of outbreaks of infected pigs were recorded in southern China, and the latest one in China occurred in Heyuan City, Guangdong Province, in 2023. Right now, 65% of the provinces in China have reported the detection of GETV, suggesting that the threat is growing in China. And in 2017 and 2018, the GETV‐infected pigs were found. Moreover, two different groups of GETV spread among pigs in Thailand [57, 58]. In South Korea, from 2003 to 2017, several pig epidemics caused by GETV were reported. Similarly, lots of infected horses were found in India in 1990 [59]. GETV was isolated from *Cx. tritaeniorhynchus* in Cambodia in 1966 (Figure 4A) [60, 61]. Improving the detection of GETV is important for finding animal epidemic situations in time and reducing damage in husbandry. In addition, it is essential to monitor the livestock industry practitioners in areas where GETV is prevalent to prevent the virus from infecting humans across species.

Figure 4Distribution of GETV. (A) Global distribution and host situation of GETV. GETV is mainly distributed in Asia and Oceania. Horses, pigs, and cattle are the natural hosts of GETV. And blue foxes and mosquitoes are found to carry GETV. (B) Distribution of GETV in China. GETV has covered more than half of the territory of China. We have drawn the spread of GETV in China by consulting the literature.

**Figure:**
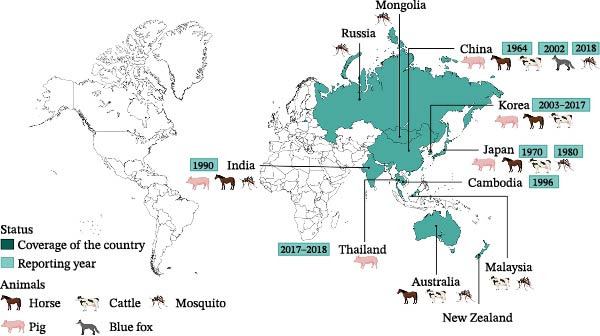
(A)

**Figure:**
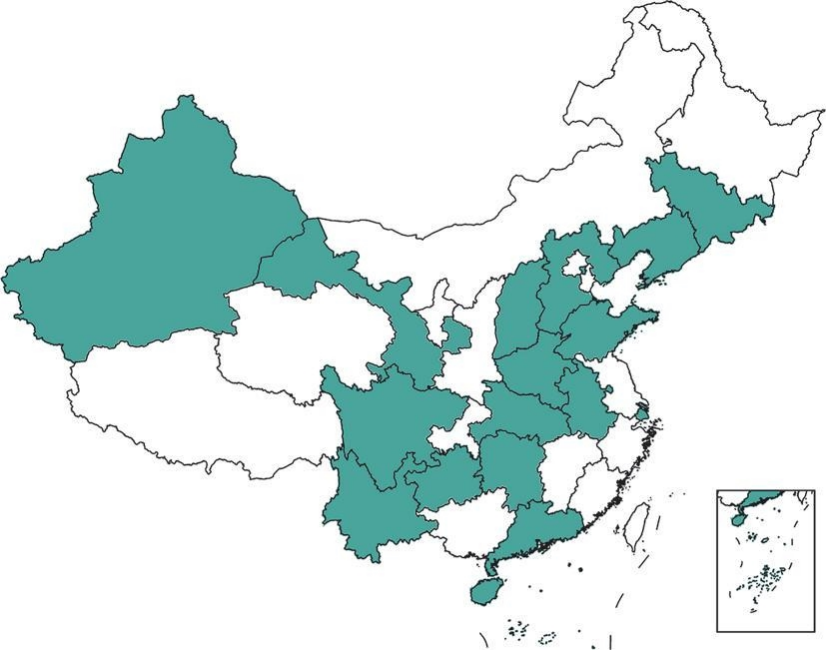
(B)

## 5. Cross‐Species Transmission

**Figure 5:**
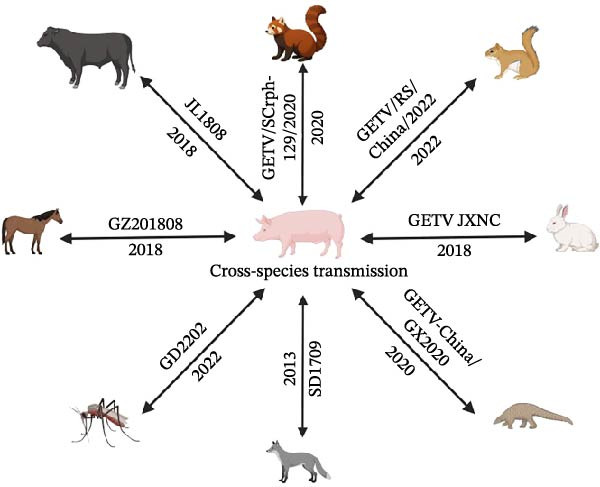
Cross‐species transmission of GETV. Current studies show that there have been multiple instances of cross‐host transmission of GETV. Researches indicate that there is cross‐species transmission between GETV’s other hosts (foxes, red pandas, horses, *Culex tritaeniorhynchus*, pangolins, rabbits, red‐bellied squirrels, and beef cattle) and pigs.

There has been an increase in the types of hosts that have become infected, and the spectrum of infections has expanded. In 2013, GETV was isolated from two infected and six deceased foxes. A subsequent sequence comparison revealed that the isolated strain (SD1709) shared 99.60% homology with the porcine GETV strain (HuN1), which was isolated from Hunan Province in 2017. GETV was identified in the deceased red panda (*Ailurus fulgens*), and the virus strain (GETV/SCrph129/2020) was detected in these red pandas in Sichuan Province. This particular strain exhibited the closest phylogenetic relationship to GETV strains originating from pigs within the province, with sequence homology ranging between 99.68% and 99.75% [62]. The GETV outbreak occurred at an equestrian training center in Guangdong Province in 2018. The comparison of the genetic sequences revealed that the strain (GZ201808) isolated in this outbreak exhibited a high similarity with the 2017 porcine‐origin GETV strain (AH9192) in China [56]. Furthermore, serum samples from beef cattle in Northeast China were found to contain GETV‐specific gene fragments. Following gene sequencing and comparison, the bovine‐derived strain (JL1808) exhibited high nucleotide sequence homology with the porcine‐derived GETV strain (HuN1) isolated in Hunan Province [63]. The Group IV GETV was detected from a pangolin in 2020, which is a new host to be found first in Guangxi Province, China. This new strain, named GETV‐China/GX2020, is highly homologous to the 2017 Thailand strain [64]. A new GETV strain named GETV JXNC was obtained from domestic rabbits in Jiangxi Province, which had the closest genetic relationship with a swine GETV strain HNNY1 in 2016, China. In 2022, GETV was first discovered in the deceased wild red‐bellied squirrel from Fujian Province, and the whole genome sequence of the virus was successfully obtained, named as strain GETV/RS/China/2022. The sequence was highly similar to the isolation GETV/SW/Thailand/2017 from domestic pigs, and the nucleotide identity of the whole genome reaches 99.90%. Besides, the GETV/RS/China/2022 strain belongs to the rare GETV Group II derivatives, indicating the expansion of the GETV host spectrum and the importance of wild animals to the evolution and spread of GETV. In the same year, the GETV GD2202 strain was isolated from *Cx. tritaeniorhynchus*. The sequence homology analysis demonstrated that the E2 gene of the GD2202 strain exhibited 99.30% nucleotide similarity with the locally isolated porcine‐derived pathogenic strains GDFS2‐2018 and GDFS9‐2018 while exhibiting 99.80% nucleotide similarity with the Henan porcine isolates HNNY‐1, HNPDS‐1, and HNPDS‐2. This finding suggests that GETV can efficiently transmit between different hosts (Figure 5) [53].

## 6. Novel Interventions

Due to the absence of understanding of the infection mechanism of GETV, there are still few antiviral treatments and potential therapeutic approaches for this virus.

Research has demonstrated that the testing of the recombinant rGECGFP with the known inhibitor ribavirin verifies that it facilitates antiviral testing against GETV. Consequently, it will be useful in the screening of potential antiviral compounds [65].

Heparin sulfate (HS) has been identified as a novel attachment factor for GETV, and the Lys253Arg mutation has been shown to enhance attachment efficiency between GETV and glycosaminoglycans (GAGs), thereby facilitating clearance of GETV from the bloodstream and reducing viral virulence. A specific residue on the E2 glycoprotein has been identified as being crucial for viral adsorption to cultured cells and pathogenicity in vivo. Substitution of residue 253 of this protein with lysine has been shown to result in reduced virulence in the infected mouse model. It is evident that the GETV E2 glycoprotein residue 253 is a pivotal site for HS binding, and this site exerts a substantial influence on the virus’s infectivity and virulence [66].

## 7. Vaccine

Previously, a whole‐virus formalin‐inactivated combined vaccine for Japanese encephalitis virus (JEV) and GETV was available for use in racehorses [67]. And the vaccine is built by the GETV strain MI‐110. There is some research investigating the correlation between the titers of antibodies in infected racehorses and their vaccination histories. The results indicate that the infected racehorses with the vaccination exhibit virus neutralization (VN) antibody titers below the detection limit and a relatively rapid decline in virus‐neutralizing antibodies [68]. Despite the vaccination, some GETV outbreaks in racehorses have been reported in Japan, indicating that the vaccine is not effective [69]. In 1990, the attenuated strain KV/VT, which had been obtained through continuous passage at low temperatures, demonstrated excellent attenuation effects in newborn piglets, adult pigs, and pregnant sows [70]. Furthermore, it has been demonstrated to elicit a robust immune response, thus paving the way for its potential utilization in developing live attenuated vaccines for pigs. GETV‐3ΔS2‐CM1, a live attenuated vaccine candidate, has a reduction in Nsp3 and substitutions in the capsid protein. It displays strong immunogenicity and stability. It is displayed to confer passive protection to piglets born to immunized sows. The chimeric virus constructed by GETV‐3ΔS2‐CM1 as the template retains the attenuated phenotype and has high immunogenicity, emphasizing its potential as a promising vaccine candidate [71]. In China, the protection rate of the inactivated vaccine with oil emulsion prepared from the isolate strain GETV‐JS18 of swine is 100% [72], and the average neutralization antibody produced by the vaccine after immunization reaches (9.8 ± 0.8) log2, and the high antibody level is maintained for at least 7 months. It can provide a reference for future vaccine development [73].

## 8. Treatment for GETV Infection

At present, there are some promising candidate drugs for treatment and prevention. The main treatment measures include giving antiviral drugs, symptomatic treatment, and strengthening nutritional support, with the aim of alleviating or controlling symptoms such as fever and limb edema. In the acute stage and early recovery stage, it is helpful for animals to rest fully. *Scutellaria baicalensis Georgi*, as a widespread Chinese medicine, has the effect of clearing away heat and toxic materials. The primary active ingredients in *S. baicalensis Georgi* are flavonoids, terpenoids, and volatile oil. Scutellarin, baicalin, and wogonin are the key active substances in the extract of *S. baicalensis Georgi* (ESG) [74]. Among these components, baicalin possesses many biological functions, including anti‐inflammatory, antiviral, antibacterial, and antitumor activities [75]. Some researches show that ESG can reduce the cytopathic effects caused by GETV in BHK‐21 cells. Meanwhile, it can alleviate virus replication and the E2 protein expression. During the viral adsorption process, the inhibition rate of replication efficiency reaches 95.08% when the concentration of ESG is 10 μg/mL. However, the inhibitory effects are not significant in both the pretreatment and postentry stages. Furthermore, the vivo studies display that the peak viral load of mice with GETV is reduced, and the duration of viremia is shortened after ESG treatment. Also, these compounds (baicalin and baicalein) and the active site of the E2 protein have strong binding affinities by molecular docking simulations [76]. Nevertheless, several studies have indicated that *S. baicalensis Georgi* possesses potential toxicity. When the dose of ethanol ESG is 2500 mg/kg, rats exhibit inflammatory changes in their liver tissue. High doses of injectable preparations may result in hypothermia and muscle soreness [77]. *S. baicalensis Georgi* has the potential to be a vaccine candidate for GETV, but related research is scarce and still in the early stage, and the inhibitory effects on infection mechanisms are still unclear.

## 9. Conclusions

Nowadays, GETV has been reported in different countries and regions, and the spread range is increasing. Besides, more species of animals are found to carry GETV, indicating that GETV has the potential to result in greater harm in husbandry. Furthermore, numerous issues require urgent resolution in the in‐depth study of dynamic changes, viral pathogenic mechanisms, and vaccine development. Although GETV has not yet resulted in any obvious symptoms, it is essential to implement a monitoring program for people engaged in animal husbandry to prevent cross‐species transmission to humans. The investigation of the pathogenic mechanisms constitutes the theoretical foundation for the development of effective pharmaceuticals and control measures. Enhancing vaccine development and application is also a priority area to reduce the threat posed by GETV.

## Funding

This study was supported by the National Natural Science Foundation of China (Grant 32002312] and the Science and Technology Project of Wenzhou, Zhejiang, China (Grant Y2023024).

## Conflicts of Interest

The authors declare no conflicts of interest.

## Data Availability

All data generated through the review process are presented within the manuscript.
